# Cardiac Complications of Hypertensive Emergency: Classification, Diagnosis and Management Challenges

**DOI:** 10.3390/jcdd9080276

**Published:** 2022-08-17

**Authors:** Mohammed A. Talle, Ellen Ngarande, Anton F. Doubell, Philip G. Herbst

**Affiliations:** 1 Department of Medicine, Division of Cardiology, Faculty of Medicine and Health Sciences, Stellenbosch University and Tygerberg Academic Hospital, Cape Town 7505, South Africa; 2 Department of Medicine, Faculty of Clinical Sciences, College of Medical Sciences, University of Maiduguri and University of Maiduguri Teaching Hospital, Maiduguri 600004, Nigeria

**Keywords:** hypertensive emergency, epidemiology, pathophysiology, cardiac acute hypertension-mediated organ damage, myocardial injury, diagnosis, classifications

## Abstract

While mortality in patients with hypertensive emergency has significantly improved over the past decades, the incidence and complications associated with acute hypertension-mediated organ damage have not followed a similar trend. Hypertensive emergency is characterized by an abrupt surge in blood pressure, mostly occurring in people with pre-existing hypertension to result in acute hypertension-mediated organ damage. Acute hypertension-mediated organ damage commonly affects the cardiovascular system, and present as acute heart failure, myocardial infarction, and less commonly, acute aortic syndrome. Elevated cardiac troponin with or without myocardial infarction is one of the major determinants of outcome in hypertensive emergency. Despite being an established entity distinct from myocardial infarction, myocardial injury has not been systematically studied in hypertensive emergency. The current guidelines on the evaluation and management of hypertensive emergencies limit the cardiac troponin assay to patients presenting with features of myocardial ischemia and acute coronary syndrome, resulting in underdiagnosis, especially of atypical myocardial infarction. In this narrative review, we aimed to give an overview of the epidemiology and pathophysiology of hypertensive emergencies, highlight challenges in the evaluation, classification, and treatment of hypertensive emergency, and propose an algorithm for the evaluation and classification of cardiac acute hypertension-mediated organ damage.

## 1. Introduction

Systemic hypertension is the most prevalent non-communicable disease and remains the leading preventable cause of premature death globally, accounting for more than 50% of cases of myocardial infarction, heart failure, and stroke [1]. Since 1990, the number of people living with systemic hypertension has doubled across the world, with low- and middle-income-countries (LMICs) accounting for most of this increase. Globally, there were roughly 1.4 billion people with systemic hypertension in 2010, and this is projected to exceed 1.6 billion by the year 2025 [2]. Approximately 1.04 billion (75%) of the global population of people with hypertension reside in LMICS [2]. South Africa has a hypertension prevalence of 35% and has the highest burden of uncontrolled hypertension amongst countries of sub-Saharan Africa [3]

The most common acute complication of systemic hypertension leading to emergency room visits is hypertensive emergency. Hypertensive emergencies represent a heterogenous group of disorders characterized by (1) acute severe blood pressure (BP) elevation, often ≥180/120 mmHg, (2) acute hypertension-mediated organ damage, and (3) the need for a prompt but contextual, system-specific lowering of the BP to avert catastrophic outcomes [4]. The organs commonly affected by acute hypertension-mediated organ damage include the heart and aorta, brain, kidneys, and retina. Concurrent occurrence of acute hypertension-mediated organ damage in multiple organs has been demonstrated, suggesting a common pathophysiologic mechanism across vascular beds [5]. Patients with severe BP elevation without evidence of acute hypertension-mediated organ damage are categorized as having hypertensive urgency, and this, along with hypertensive emergency, constitutes the syndrome of hypertensive crisis. However, the European Society of Cardiology (ESC) Council on hypertension recently proposed replacing the term hypertensive urgency with “uncontrolled hypertension”, therefore rendering the umbrella term hypertensive crisis (hitherto used to describe hypertensive emergency and hypertensive urgency) unnecessary [4].

Cardiac complications are the most prevalent acute hypertension-mediated organ damage in hypertensive emergencies. The three major cardiac acute hypertension-mediated organ damage syndromes include acute heart failure/cardiogenic pulmonary oedema, acute coronary syndrome (ACS), and less commonly, acute aortic syndrome (primarily acute aortic dissection) [6,7,8,9]. Mortality in hypertensive emergency is substantially elevated, especially among patients admitted into coronary care units when compared to patients without hypertensive emergencies [10]. One of the prognostic factors for major adverse cardiac events (MACE) and cerebrovascular events in patients with hypertensive emergency is raised cardiac troponin levels, with or without proven ACS [11,12].

Despite numerous studies and reports on cardiac complications of hypertensive emergencies, uncertainties remain. Different studies report varying prevalence rates, reflecting the heterogenous nature of the studies and potentially, selection bias. The use of terminology in the classification and reporting of hypertensive emergency has been inconsistent across studies (e.g., the use of acute heart failure and pulmonary oedema interchangeably in some studies, and separately in others). Despite the overwhelming evidence of the prognostic implications of elevated cardiac troponin and subclinical cardiac injury, acute myocardial injury is not considered as acute hypertension-mediated organ damage. Presently, no robust system exists for risk stratification to promptly identify subgroups at a high risk of adverse cardiovascular and renal outcomes that could pre-emptively guide future management.

In this narrative review, we aimed to: (1) give an overview of the epidemiology and pathophysiologic mechanisms of hypertensive emergency, (2) highlight the prevalence of cardiac complications of hypertensive emergency, (3) identify challenges in the evaluation, identification, classification and reporting of cardiac complications of hypertensive emergency, (4) propose an algorithm for the evaluation and classification of cardiac complications of hypertensive emergency, including the routine assay of cardiac troponin, and (5) make recommendations for the future.

## 2. Epidemiology

Although the availability of effective and well-tolerated antihypertensive medications has significantly improved outcomes in patients with hypertensive emergencies, the incidence remains unchanged [13,14]. An estimated 2–3% of hypertensive patients will develop hypertensive emergency in their lifetime [15,16]. Data on gender differences in patients with hypertensive emergency have been inconsistent, with some studies showing a predominance of males [7,8,17,18], and others showing comparable prevalence in males and females [6,15,19]. Similarly, reports of age distribution compared with patients having acute severe hypertension without acute hypertension-mediated organ damage has been contradictory [9].

Studies report a varying prevalence of cardiac acute hypertension-mediated organ damage, depending on demographics and comorbidities, among others; however, cardiac involvement predominates in most of the studies, with a cumulative prevalence ranging from 3.6 to 91% (Table 1). Reasons for this marked variation in prevalence include: (1) selection bias due to preferential referrals to specialized centers; (2) variation in the exclusion criteria applied; (3) selective use of cardiac troponin assays resulting in underdiagnosis of atypical cases of myocardial infarction; (4) the non-inclusion of patients managed at primary and secondary care levels without referral to tertiary centers where most of the studies were carried out. A recent systematic review reported a composite prevalence of 52% for cardiac involvement in patients with hypertensive emergencies [9]. Epidemiology of the different cardiac acute hypertension-mediated organ damage is further discussed in the section for specific cardiac complications of hypertensive emergency.

## 3. Pathophysiology

The exact pathophysiologic mechanisms of hypertensive emergency remain incompletely understood. However, a sudden rise in BP serves as a common denominator underlying the various forms of acute hypertension-mediated organ damage, and most hypertensive emergencies occur in people with pre-existing hypertension [4]. Although triggers for the surge in BP are also not clearly understood, nonadherence to antihypertensive medications, stress, and increased salt intake have been identified as major risk factors [6]. Three intrinsically interwoven processes operating in concert play an important role in the pathophysiology. These include the failure of vascular autoregulation, endothelial dysfunction, and activation of the renin angiotensin aldosterone system (RAAS).

The principal function of vascular autoregulation is to ensure uninterrupted blood flow to vital organs during fluctuations in BP and perfusion pressure, and this is accomplished via the appropriate modification of the peripheral vascular resistance (PVR) [24,25]. Vascular resistance is constantly modified by metabolic, myogenic, and endothelial modulators acting in concert [26]. During increased BP and perfusion pressure, vascular resistance increases to mitigate hyper perfusion-induced organ injury, while in the face of hypotension and reduced perfusion pressure, vasodilation results in reduced vascular resistance to maintain flow to vital organs. In hypertensive emergency, a surge in BP and increased intravascular shear stress results in the disruption of vascular autoregulation and endothelial damage. This causes increased vascular permeability, perivascular oedema, exposure of subendothelial contents to circulating blood, and thrombogenesis [27]. The ensuing microvascular damage and thrombotic occlusion results in hemolysis, hypoperfusion, release of cytokines and proinflammatory molecules, ischemia, and activation of the RAAS [27,28].

Heightened activation of the RAAS and increased levels of angiotensin II is nearly ubiquitous in patients with hypertensive emergency and correlates with the extent of microvascular damage [28]. Angiotensin II is a potent mediator of vasoconstriction, inflammation, endothelial dysfunction, remodeling, and vascular fibrosis, and stimulates the secretion of aldosterone [29]. In addition to its principal role of volume expansion and BP maintenance, aldosterone causes cardiovascular and renal inflammation, fibrosis, and remodeling [30]. Recent studies demonstrated the expression of mineralocorticoid receptors in endothelial and vascular smooth muscle cells, resulting in aldosterone-induced vascular inflammation, fibrosis, and remodeling, as well as vascular smooth muscle cell hypertrophy and proliferation [31,32,33]. RAAS also exerts stimulatory effects on the cerebral sympathetic nervous system and potentiates the release of norepinephrine [34]. Increased levels of norepinephrine are associated with natriuresis, volume contraction, and the activation of RAAS, thus, establishing a vicious cycle.

Fibrinoid necrosis of small muscular arteries and arterioles, characterized by medial smooth muscle cell necrosis and the focal deposition of proteinaceous material occurs in malignant hypertension, a form of hypertensive emergency [35]. This is succeeded by proliferative endarteritis, characterized by intimal thickening, hyperplasia of the intimal fibroblasts, generation of collagen fibers, and atrophy of the media. Fibrinoid necrosis and proliferative endarteritis are considered the histological hallmark (but not pathognomonic) of malignant hypertension, and both may result in impaired perfusion and ischemia [35]. These changes have been demonstrated in various organs including the kidney, brain, intestine, and pancreas [36]. In one proof-of-concept study, the intravenous injection of angiotensin II in an experimental model of hypertension resulted in increased endothelial permeability and necrosis of cardiac myocytes and intramyocardial arterioles, with sparing of the epicardial coronary arteries [37].

**Figure 1:**
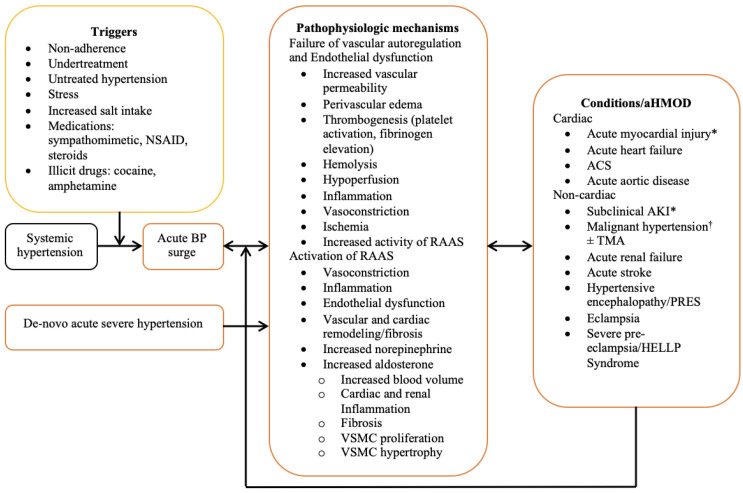
Summary of the pathophysiologic processes in acute hypertension-mediated organ injury. ACS; acute coronary syndrome; AKI, acute kidney injury; aHMOD, acute hypertension-mediated organ damage; BP, blood pressure; HELLP, hemolysis, elevated liver enzymes, low platelets; NSAID, nonsteroidal anti-inflammatory drug; PRES, posterior reversible encephalopathy syndrome; RAAS, renin–angiotensin–aldosterone system; TMA, thrombotic microangiopathy; VSMC, vascular smooth muscle cell; * Not listed as acute hypertensive target organ damage in guidelines; † presence of retinal exudates, hemorrhage ± papilledema.

The constellation of pathophysiologic events described above does not occur in any preferential order, but rather, evolves concurrently in a variety of sequences with overlaps and widespread involvement of the vascular beds across various organs. The combined effects of autoregulatory failure, endothelial dysfunction and RAAS activation establishes a vicious cycle of BP elevation and progressively worsening acute hypertension-mediated organ damage. A summary of the pathophysiological mechanisms is presented in Figure 1.

## 4. Specific Cardiac Complications of Hypertensive Emergency

The different cardiac complications of hypertensive emergency are presented in Table 2.

### 4.1. Acute Heart Failure and Cardiogenic Pulmonary Edema

Acute heart failure (including cardiogenic pulmonary edema) is the most common cardiac complication in hypertensive emergency with a prevalence ranging from 21.1 to 58% (Table 1). A recent systematic review reported a prevalence of 32% [9]. 

Most patients presenting with hypertensive emergency have preexisting hypertension, and as many as 83% have left ventricular hypertrophy (LVH) and diastolic dysfunction [18]. An acute rise in BP results in increased ventricular–vascular uncoupling and elevated left ventricular filling pressure [38]. This forms the basis of pulmonary edema and heart failure with a preserved ejection fraction, the most common form of heart failure in hypertensive emergencies. Reversible left ventricular systolic heart failure and left ventricular dilatation have also been reported [39]. Takotsubo cardiomyopathy and impaired systolic function occurred in patients with Pickering syndrome and hypertensive crisis, which was associated with postoperative volume expansion and phaeochromocytoma [40,41,42].

Clinical features include a cough with frothy (pinkish) sputum, dyspnea, orthopnea, tachycardia, and elevated BP. Other findings are a heaving (often un-displaced) cardiac apex, S_3_ or summation gallop, and bibasilar crackles. Features of pulmonary congestion, cardiomegaly, and a widened mediastinum (due to aortic disease) may be visible on plain chest radiograph. However, subtle forms of cardiomegaly may not be apparent on conventional posterior-to-anterior chest X-ray. Echocardiography (the most frequently used diagnostic tool in heart failure) is useful in demonstrating cardiac morphology, function, and elevated left ventricular filling pressure. Reversible subclinical left ventricular systolic dysfunction has been reported with speckled-tracking echocardiography [43].

The lowering of BP is associated with a prompt improvement of symptoms and outcomes in patients with acute hypertensive heart failure [44]. The ESC Council on hypertension recommends the immediate lowering of systolic BP to 140 mmHg or lower using intravenous medications, with close hemodynamic monitoring in an intensive care unit [4]. Drugs of choice include Nitroprusside and Nitroglycerin, which have the advantage of acutely reducing ventricular pre- and after-load, and this effect can be augmented when combined with a loop diuretic. Overzealous diuresis, however, may be counterproductive since the pulmonary edema in hypertensive emergency is driven by elevated left ventricular end-diastolic pressure and not volume overload. Urapidil, a selective post-synaptic alpha-1 antagonist can be used as an alternative and has the advantage of reducing PVR without increasing the heart rate.

Studies on the outcome of acute hypertensive heart failure are conflicting. While some studies found hypertension to be a predictor of low-risk for 30-day mortality and readmission [45], reports from the STAT (Studying the Treatment of Acute Hypertension) registry revealed increased in-hospital mortality, intensive care unit admission, likelihood of readmission, and prolonged hospitalization among patients with acute hypertensive heart failure [46]. There is overwhelming evidence linking cardiac troponin elevation to an increased risk of exacerbation, readmission, cardiac, and total mortality in patients with acute heart failure [47,48]. However, cardiac troponin is not routinely measured in patients with hypertensive emergency and acute heart failure. Routine cardiac troponin assay in patients with hypertensive emergency can promptly identify atypical ACS masquerading as acute heart failure/cardiogenic pulmonary edema, as well as a subgroup of patients at higher risk for MACE [49].

### 4.2. Acute Coronary Syndrome

Acute coronary syndrome is an umbrella term encompassing ST-elevation myocardial infarction, non-ST-elevation myocardial infarction and unstable angina. The prevalence of myocardial infarction in patients with hypertensive emergency ranges from 1 to 59.5% (Table 1). These varying prevalence rates underscore the heterogeneity in the studied populations, the study settings, definitions of myocardial infarction applied, and the selective use of the cardiac troponin assay. A recent systematic review reported a composite prevalence of 18% for ACS among patients with hypertensive emergencies [9].

Endothelial injury and intravascular thrombosis associated with hypertensive emergency could result in Type 1 myocardial infarction, especially in patients with pre-existing coronary artery disease. One study reported a prevalence of 76.5% for coronary artery disease among patients with hypertensive emergency and elevated troponin who underwent coronary angiography [23]. The structural (LVH) and microvascular alterations inherent to long-standing hypertension provide a veritable substrate for myocardial demand–supply mismatch, setting the stage for impaired myocardial perfusion and Type 2 myocardial infarction. Ventricular–vascular uncoupling and the augmentation index associated with increased PVR in patients with hypertensive emergency increases the risk for Type 2 myocardial infarction [50]. The recently published DEMAND MI (Determining the Mechanism of Myocardial Injury and Role of Coronary Disease in Type 2 myocardial infarction) study found a coronary artery disease prevalence of 68% among their cohort with Type 2 myocardial infarction, with 30% demonstrating obstructive disease [51]. Rarely, dissection of the aorta can affect the coronary circulation (commonly the right coronary artery) resulting in myocardial ischemia and infarction [52].

Diagnosis of myocardial infarction is established in the presence of an elevated cardiac troponin levels above the 99th percentile of the upper reference limit (URL) with a rising and/or falling pattern, and at least one of the following: (1) symptoms of myocardial ischemia, (2) new ischemic ECG changes, (3) development of pathological Q waves, (4) evidence of new loss of viable myocardium or regional wall motion abnormalities in a coronary distribution, and (5) demonstration of coronary thrombus [50]. Symptoms include chest discomfort, shortness of breath, cough, and diaphoresis, among others. It is important to note that myocardial ischemia/infarction can be silent following acute BP rise and may present as cardiogenic pulmonary oedema and heart failure.

The aim of BP lowering in hypertensive emergency and ACS is to reduce myocardial oxygen demand by afterload reduction without compromising left ventricular diastolic filling. The ESC Council on hypertension recommends the immediate (within 1 h) lowering of the systolic BP to 140 mmHg or lower using nitroglycerin or labetalol [4]. A combination of beta-blockers and nitroglycerine has the advantage of preventing reflex tachycardia. Urapidil, a selective postsynaptic alpha-1 antagonist with vasodilating properties, also has the advantage of not being associated with reflex tachycardia and can be used in place of nitroglycerin or nitroprusside. Despite being contraindicated [53], sublingual nifedipine is not infrequently used, especially in LMICs including countries of sub-Saharan Africa. This can cause a precipitous fall in BP resulting in stroke, the worsening of myocardial ischemia and infarction, especially in elderly patients with preexisting vascular disease or target organ damage [53,54]. Beyond the lowering of BP, ACS is managed according to standard guidelines, keeping in mind that the risk of intracerebral bleeds is increased with the use of fibrinolytics and antithrombotic medications.

The concurrence of acute severe hypertension and myocardial infarction presents a diagnostic and management challenge. Blood pressure increases could either be the consequence, or cause of acute myocardial infarction (and stroke), and spontaneous reduction in BP has been reported in patients presenting with acute myocardial infarction and severe hypertension within 6 h of admission without the use of antihypertensive medications [55]. Demonstrating evidence of parallel acute hypertension-mediated organ damage in other vascular beds (e.g., retinal hemorrhage) could favor acute severe hypertension as the cause rather than consequences of myocardial infarction.

The different subtypes of ACS have varying pathophysiological mechanisms and prognoses, and it is unclear as to what degree unstable angina (without objective evidence of cardiac injury or acute hypertension-mediated organ damage) can be regarded as true hypertensive emergency. Most of the studies reporting ACS did not provide details of the subtypes to allow for appropriate risk stratification and prognostication. Another limitation is the selective application of the cardiac troponin assays, resulting in an underdiagnosis of atypical ACS (e.g., those presenting with acute heart failure and angina equivalent).

### 4.3. Acute Myocardial Injury

The universal definition of myocardial infarction identified myocardial injury separately from myocardial infarction in the ‘continuum’ of acute ischemic heart disease [50]. Acute myocardial injury is defined as a troponin rise above the 99th percentile URL with a rising and/or falling pattern, without evidence of myocardial ischemia, including ECG changes. A sustained elevation in cardiac troponin above the 99th percentile URL (without rising or falling pattern) on serial measurements is considered a chronic myocardial injury.

Fibrinoid necrosis, proliferative endarteritis, and intravascular thrombosis (the pathological hallmarks of hypertensive emergencies) could result in myocardial injury [37,50]. However, very few studies reported myocardial injury in hypertensive emergency, with a prevalence ranging from 15 to 63% (Table 1). The true prevalence is difficult to determine from the available studies as cardiac troponin is only measured based on the presence of features of myocardial ischemia/infarction [4,56,57,58]. In one study, 63% of the cohort had elevated cardiac troponin without features of myocardial ischemia, implying subclinical myocardial injury [18]. Similarly, 60.4% of the patients with elevated cardiac troponin and acute hypertension-mediated organ damage in another study did not have ACS [12], in keeping with the diagnosis of myocardial injury.

The initial evaluation of patients with myocardial injury is aimed at identifying features of myocardial ischemia and myocardial infarction. Where there are no features of myocardial ischemia, the evaluation is focused on non-cardiovascular causes. Without doing a cardiac troponin assay, myocardial injury could go unrecognized, underscoring the need for a routine cardiac troponin assay in all patients presenting with hypertensive emergency. Presently, there are no guidelines or consensus regarding the management of patients with acute myocardial injury and treatment is tailored to managing the triggers.

Differentiating acute myocardial injury from Type 2 myocardial infarction is challenging. Each of the two conditions may represent different points along a single continuum of varying severity of cardiac complications, and they may co-exist in patients with hypertensive emergency [50,59]. The diagnosis in both requires cardiac troponin levels above the 99th percentile URL with a rising and/or falling pattern and are only differentiated based on features of myocardial ischemia. However, myocardial ischemia and infarction can present with atypical/nonspecific features or be asymptomatic (silent) [50]. Also, LVH being highly prevalent in hypertension and hypertensive emergency limits the use of ECG repolarization abnormalities in the detection of myocardial ischemia. Features of myocardial injury and Type 2 myocardial injury are summarized in Table 3.

Most studies looking at the prognostic impact of myocardial injury did not differentiate acute from chronic myocardial injury, and hypertensive emergency was not represented in the cohort. Overall, patients with myocardial injury have a poorer prognosis compared to patients without [12,49,59]. In one study, all-cause mortality rates among hospitalized patients and readmission rates at 30 days were 11 and 21%, respectively [61]. A long-term outcome study reported a 5-year mortality rate of 72.4% [49]. Although the mortality in the latter was mainly driven by non-cardiovascular causes, MACE (non-fatal myocardial infarction or cardiovascular death) occurred in 31%.

There is increasing interest and awakening towards the identification of high-risk groups among patients with myocardial injury. The troponin assessment for risk stratification of patients without acute coronary atherothrombosis (TARRACO) risk score [62] was recently formulated and showed good internal and external validity. In addition to cardiac troponin levels, variables included in the score are age, hypertension, dyspnea, anemia, and absence of chest pain. The TARRACO risk score has a total of 13 and dichotomizes patients into low-risk (scores 0–6) and high-risk (scores of 7–13) groups. MACE was reported to be five times higher in the high-risk group [62]. Further evaluation in clinical trials is, however, required to validate its prognostic value.

### 4.4. Acute Aortic Syndrome

Acute aortic syndrome comprises of acute aortic dissection, intramural hemorrhage/hematoma, a penetrating atherosclerotic aortic ulcer and aortic rupture. Acute aortic dissection (AAD) is the most prevalent acute aortic syndrome complicating hypertensive emergency. However, it is the least common cardiac complication of hypertensive emergency with a prevalence of 0 to 6.3% (Table 1). A recent meta-analysis involving nine studies found a prevalence of 2% [9]. Other forms of acute aortic syndrome have only been sparingly reported in patients presenting with malignant hypertension-range BP, and a rare occurrence of triple aortic syndrome has been reported in a single patient with hypertensive emergency [63].

The true incidence and prevalence of AAD is difficult to determine because of a high out-of-hospital mortality, diverse/overlapping clinical presentations, and misdiagnosis [64,65]. Pre-hospitalization mortality rates range from 21 to 49%, and up to 50% of in-hospital mortality occur within 24 h, mainly due to delayed and missed diagnosis [65,66]. In a population-based study involving 66 patients, misdiagnosis occurred in 71.1%, with one-third diagnosed as acute myocardial infarction [67]. 

Acute aortic dissection commonly present with an abrupt onset of chest pain (often described as tearing or ripping and radiating to the interscapular region of the back) and severe BP elevation. However, patients with cardiac tamponade (from retrograde extension into the pericardial sack or aortic rupture) may present with hypotension. Other symptoms of AAD include back pain, abdominal pain, pre-syncope/syncope, lateralizing signs (hemiparesis/hemiplegia, paraparesis/paraplegia, aphasia), and loss of consciousness [64]. Extension of type A dissection into the brachiocephalic trunk and the left subclavian artery may result in a pulse deficit and asymmetrical arm BP in up to 20% of cases [68]. Acute aortic regurgitation may result from involvement of the aortic root and/or cusps. The ostium of the coronary artery (commonly the right) may be occluded by bulging of a false lumen, intimal detachment, retrograde extension of the dissection or flail flap resulting in myocardial ischemia/infarction [69]. Diagnosis of AAD is confirmed with transesophageal echocardiography, contrast-enhanced computed tomography, or magnetic resonance imaging.

Treatment of AAD is aimed at reducing pulsatile hemodynamic stress on the aortic wall to forestall propagation of the dissection and prevent aortic rupture. The ESC Council on hypertension recommends immediate (within 20 min) lowering of systolic BP to between 100 mmHg and 120 mmHg, and the heart rate to less than 60 beats per minute using short-acting beta-blockers [4]. Although parenteral metoprolol and labetalol can be used, their long half-life can impede on the correction of hypotension if required. Attainment of a desirable BP may require the administration of nitroprusside, nitroglycerine, or nicardipine in combination with esmolol to prevent reflex tachycardia. The definitive treatment for dissection should be carried out in accordance with standard practice guidelines.

Diagnosis of AAD can be elusive, requiring a high index of suspicion. The most common misdiagnosis is acute myocardial infarction [67]. Mortality is commonly due to cardiac tamponade resulting from aortic rupture, and elevated cardiac troponin is a proven predictor of rupture especially in Stanford type A dissections [70]. Percutaneous pericardiocentesis was previously contraindicated in cardiac tamponade caused by aortic dissection [71]. However, the recent ESC guideline on pericardial disease recommends controlled pericardial drainage of small amounts of hemopericardium to maintain systolic BP at 90 mmHg and temporarily stabilize the patient [72].

## 5. Challenges in Evaluation, Classifications, and Treatment of Cardiac Complications of Hypertensive Emergencies

### 5.1. Sub-Clinical Acute Target Organ Damage

Based on current guidelines, the measurement of cardiac troponin in patients with hypertensive emergency is recommended only when there are symptoms/features of myocardial ischemia. Asymptomatic/sub-clinical myocardial injury occurs in more than one-third of patients with hypertensive emergency [13] and there is evidence for an increased risk of MACE and poor renal outcome in patients with myocardial injury [50]. Notwithstanding, current guidelines on the evaluation of hypertensive emergency do not include assessment of subclinical acute target organ damage/dysfunction. The selective use of cardiac troponin assays can result in missed and mis-diagnoses of atypical acute myocardial infarction (including silent myocardial infarction), and subclinical myocardial injury.

### 5.2. Nomenclature and Classification

The nomenclature and classification of cardiac complications of hypertensive have not been consistent. Many studies used acute heart failure and acute pulmonary oedema (or cardiogenic pulmonary oedema) interchangeably, whereas some reported the two separately.

The universal definition categorizes myocardial infarction into five types [51]. However, most studies on the cardiac complications of hypertensive emergency fall short of defining the different subtypes of ACS or myocardial infarction despite differences in their underlying pathophysiologic mechanisms and outcomes/prognosis. It is unclear as to what proportion of patients with ACS had ST-elevation myocardial infarction, non-ST-elevation myocardial infarction or unstable angina, and it remains debatable as to what extent unstable angina will be considered a true acute hypertension-mediated organ damage as defined in current guidelines.

### 5.3. Treatment

There are no randomized controlled trials to guide treatment in most cases of hypertensive emergencies, and the choice of medications, as well as the rate and magnitude of BP reduction is mainly based on expert opinion [5]. The ESC Council on hypertension recommends intravenous medications with close hemodynamic monitoring in an intensive care unit, which may not be available in low-resource settings, especially in LMICs. Evidence is emerging for the efficacy of orally administered medications in the treatment of hypertensive emergencies. In one study involving patients with malignant hypertension, the cohorts were treated with sequential administration of oral renin–angiotensin system blockers, calcium blockers, thiazide diuretic and spironolactone as required, without the need for admission into intensive care unit [19]. This cost-effective approach to treatment will appeal to LMICs with limited resources.

Presently, there are no well-validated systems for risk stratification or consensus regarding the best management options for high-risk groups, including patients with subclinical target organ damage. Based on the results of the DEMAND MI study, patients with suspected Type 2 myocardial infarction should be subjected to routine assessment of their coronary arteries (invasive or computed tomography) and be given the benefit of evidence-based treatments to improve outcomes [52]. This is, however, based on a single study and there is still a need for further clinical trials including patients with hypertensive emergency, to determine outcomes.

### 5.4. Biomarkers of Subclinical Myocardial Injury

Cardiac troponin is undoubtedly the best indicator of myocardial injury. However, new biomarkers of myocardial injury are being increasingly identified. Cardiac myosin-binding protein C is a novel biomarker of myocardial injury that is more sensitive and has the advantage of rising and falling more quickly than cardiac troponin [73]. This allows for more efficient tracking of the onset and resolution of acute hypertension-mediated organ damage, especially following intervention. Cardiac myosin-binding protein C has not been studied in hypertensive emergencies. It may be worthwhile to explore this and other biomarkers of myocardial injury including cardiac magnetic resonance imaging in the diagnosis of subclinical cardiac acute hypertension-mediated organ damage.

## 6. Recommendations for the Future 

The following recommendations are aimed at addressing some of the challenges highlighted above:

i. Subclinical acute hypertensive target organ damage/dysfunction should be actively sought and added to the categories of acute hypertension-mediated organ damage (Table 2). This should include subclinical cardiac (acute myocardial injury), renal (subclinical acute kidney injury) and brain injury.
ii. There is a need for consistency in the nomenclature and classification of acute hypertension-mediated organ damage. Acute heart failure should be used instead of cardiogenic pulmonary edema. The different types of myocardial infarction should be categorically identified as defined in the universal definition of myocardial infarction and included in studies reporting cardiac complications of hypertensive emergency.
iii. There is the need for a properly designed study to: (i) accurately determine the true burden of acute hypertension-mediated organ damage in patients with hypertensive emergencies; (ii) determine the markers and long-term outcomes of subclinical acute hypertension-mediated organ damage; (iii) provide evidence-based strategies for immediate and long-term management of the different forms of acute hypertension-mediated organ damage; (iv) validate the use of oral medications in the treatment of hypertensive emergency; (v) develop well-defined strategies for the evaluation and management of acute myocardial injury and Type 2 myocardial infarction in patients with hypertensive emergencies.

**Figure 2:**
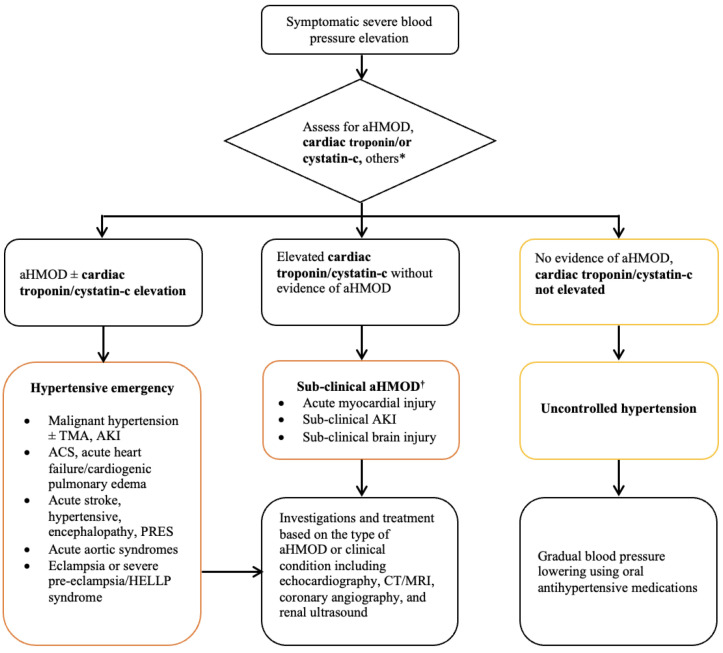
Algorithm for evaluation and classification of hypertensive emergencies. ^†^ Not listed as target organ damage in guidelines. ACS, acute coronary syndrome; AKI, acute kidney injury; aHMOD, acute hypertension-mediated organ damage; HELLP, hemolysis elevated liver enzymes or low platelets; PRES, posterior reversible encephalopathy syndrome; TMA, thrombotic microangiopathy. * Others—hemoglobin, platelet, lactic dehydrogenase, haptoglobin, creatinine, sodium, potassium, quantitative urinalysis for protein, urine sediments, ECG, chest X-ray, fundoscopy. Modified from van den Born et al. [5].

A proposed algorithm for evaluation and classification of acute severe rise in blood pressure and hypertensive emergency is presented in Figure 2.

## Figures and Tables

**Table 1:** Prevalence of cardiac acute hypertension-mediated organ damage in hypertensive emergencies.

Author, Year, Country	Design	AHF (%)	AMI (%)	AAS (%)	Cumulative (%)	NIMI (%)	Comments
Fragoulis [6], 2021, Greece	Prospective	58	22.6	2	82.6	NR	National cardiac referral centre registry data. Potential for bias towards cardiac complications.
Rubin [18], 2019, France	Prospective	31	NR	NR	31%	63	Excluded myocardial infarction from their cohorts and 63% had elevated troponin while 83% had left ventricular hypertrophy.
Zampaglione [15], 1996, Italy	Prospective	36.8	12	2	50.8	NR	Cerebral infarction was the most common acute hypertension-mediated organ damage. However, composite of cardiac complications occurred in 50.8%.
Kim [12], 2022, Korea	CS	NR	40.5	NR	40.5	60.4	Focused on prognostic role of cardiac troponin in acute severe hypertension. Elevated (occurred in 41.6%) and detectable (occurred in 36.5%) cardiac troponin associated with higher mortality at 3 years.
Guiga [20], 2017, France	CS	37.4	13.8	1.8	53	NR	Reported higher mortality in hypertensive emergency than hypertensive urgency (12.5 vs. 1.8%).
Salvetti [8], 2021, Italy 2008 data 2015 data	Prospective	34 37.5	25 25	1 0.5	60 63	NR NR	Excluded resuscitated cardiac arrest and patients requiring urgent cardiac catheterization.
Pacheco [7], 2103, Mexico	Prospective	25.2	59.5	6.3	91	NR	Their cohorts composed of a high-risk group admitted into coronary care unit. Reported high rate of acute coronary syndrome and acute aortic syndrome.
Martin [17], 2004, Brazil	Retrospective	25	13	0	33	NR	Reported unstable angina (5%) separately from myocardial infarction (8%).
Vilela-Martin [21], 2011, Brazil	CS	30.7	25.1	3.5	47.2	NR	Reported unstable angina (12.1%) separately from myocardial infarction (13%).
Nkoke [19], 2020, Cameroon	CS	44.6	3.6	0	48.2	NR	Myocardial infarction occurred in 3.6% of their cohorts. Low rate of detection of myocardial infarction may be related to lack of facilities including low rates of ECG and cardiac troponin assay.
Acosta [22], 2020, USA	Retrospective	NR	1	0	1	15	Assessed acute myocardial injury using serial cardiac troponin assay. Excluded acute coronary syndrome from their cohorts.
Pattanshetty [23], 2012, USA	Retrospective	20.5	11.7	2.3	34.5	NR	Obstructive coronary artery disease present in 76.5% of their cohorts with elevated cardiac troponin that had angiogram.

AAS, acute aortic syndrome; AHF, acute heart failure; AMI, acute myocardial infarction; CS, cross-sectional; NIMI, non-ischemic myocardial injury; NR, not reported; USA, United State of America.

**Table 2:** Cardiac complications of hypertensive emergency.

**Acute hypertension mediated-organ damage**
Acute heart failure/acute pulmonary edema *
Acute coronary syndrome *
ST-elevation myocardial infarction
Non-ST-elevation myocardial infarction
Unstable angina
Acute aortic syndrome
Acute aortic dissection *
Intramural hemorrhage/hematoma
Penetrating atherosclerotic aortic ulcer
Aortic aneurysm
Aortic rupture
**Sub-clinical cardiac target organ injury ^§^**
Acute myocardial injury

* Commonly reported cardiac complications; ^§^ Not included as a complication in guidelines.

**Table 3:** Comparison of myocardial injury with Type 2 myocardial infarction (modified from [60]).

	Myocardial Injury	Type 2 Myocardial Infarction	Comment
**Definition**	At least 1 cardiac troponin concentration above the 99th percentile URL without features of myocardial ischemia/infarction	Rise and/or fall in cardiac troponin level with at least 1 value above the 99th percentile URL with at least one of the following: (1) Symptoms of myocardial ischemia (2) New ischemic ECG changes (3) Development of pathological Q waves (4) Imaging evidence of new loss of viable myocardium or new ischemic RWMA.	• Signs and/or symptoms of myocardial ischemia/myocardial infarction may be atypical. • LVH limits the use of ECG repolarization abnormalities in detection of myocardial ischemia.
**Mechanism of troponin rise**	Myocardial strain, inflammation, apoptosis, and cell injury.	Myocardial infarction due to mismatch in myocardial oxygen supply–demand in the absence of atherothrombotic event.	Pathophysiologic mechanisms in hypertensive emergency involve inflammation and demand-supply mismatch [27,50]. Myocardial injury and Type 2 myocardial infarction can occur in hypertensive emergencies.
**Management strategies**	Undefined	Undefined	
**Coronary anatomy and left ventricular function**	Not systematically studied	CAD in 68% (obstructive in 30%), LVSD in 34% [51].	Both predict the presence of coronary artery disease and MACE.
**Outcomes**			The similarities in outcome measures reflects shared pathophysiologic mechanisms.
In-hospital all-cause [61]	~11%	~9%	
Post-discharge 30-day [61]	~7%	~4%	
5-year all-cause [49]	~72%	~63%	
5-year MACE [49]	~31%	~30%	
30-day readmission [61]	~21%	~21%	

CAD, coronary artery disease; ECG, electrocardiogram; LVH, left ventricular hypertrophy; LVSD, left ventricular systolic dysfunction; MACE, major adverse cardiovascular event; RWMA, regional wall motion abnormality; URL, upper reference limit.

## Data Availability

Not applicable.
